# Effects of transcranial direct current stimulation in combination with motor practice on dexterous grasping and manipulation in healthy older adults

**DOI:** 10.1002/phy2.255

**Published:** 2014-03-20

**Authors:** Pranav J. Parikh, Kelly J. Cole

**Affiliations:** ^1^ Motor Control Laboratories Department of Health and Human Physiology University of Iowa Iowa City Iowa 52242; ^2^ Present address: Pranav J. Parikh, Neural Control of Movement Laboratory Arizona State University Tempe Arizona 85287

**Keywords:** Aging, cortical stimulation, force, prehension

## Abstract

Transcranial anodal stimulation (tDCS) over primary motor cortex (M1) improves dexterous manipulation in healthy older adults. However, the beneficial effects of anodal tDCS in combination with motor practice on natural and clinically relevant functional manual tasks, and the associated changes in the digit contact forces are not known. To this end, we studied the effects of 20 min of tDCS applied over M1 for the dominant hand combined with motor practice (MP) in a sham‐controlled crossover study. We monitored the forces applied to an object that healthy elderly individuals grasped and manipulated, and their performances on the Grooved Pegboard Test and the Key‐slot task. Practice improved performance on the Pegboard test, and anodal tDCS + MP improved retention of this performance gain when tested 35 min later, whereas similar performance gains degraded in the sham group after 35 min. Interestingly, grip force variability on an isometric precision grip task performed with visual feedback of precision force *increased* following anodal tDCS + MP, but not sham tDCS + MP. This finding suggests that anodal tDCS over M1 might alter the descending drive to spinal motor neurons involved in the performance of isometric precision grip task under visual feedback leading to increased fluctuations in the grip force exerted on the object. Our results demonstrate that anodal stimulation in combination with motor practice helps older adults to retain their improved performance on a functionally relevant manual task in healthy older adults.

## Introduction

After the seventh decade of life, healthy adults experience difficulty in performing dexterous manual tasks such as preparing meals, tying shoelaces, inserting key in its hole, buttoning‐unbuttoning clothing (Desrosiers et al. 1999; Smith et al. 1999). Several intervention techniques such as practice, strength training, peripheral, and cortical stimulation, to mention a few, have been proposed to either improve fine manual performance in older adults or retard its deterioration. A single 20‐min session of anodal transcranial direct current stimulation (tDCS) over primary motor cortex (M1) improved manual performance in older adults (Hummel et al. 2010). When anodal tDCS was delivered in association with motor practice better behavioral outcomes were achieved in comparison with standalone therapy in young adults (Reis et al. 2008, 2009; Galea and Celnik 2009; Bolognini and Ro 2010; Stagg et al. 2011). More recently, anodal tDCS in combination with motor practice facilitated acquisition and retention of learning on a complex finger‐tapping task in older adults (Zimerman et al. 2013). This raises an important question whether anodal tDCS *when combined with motor practice* can improve motor performance on natural and functionally meaningful object manipulation tasks in older adults.

Decline in manual dexterity in older age has been associated with behavioral slowing, and impaired control of finger forces applied to the object during grasp and manipulation (Salthouse 1988; Smith et al. 1999; Enoka et al. 2003; Diermayr et al. 2011). Older adults demonstrate an impaired control over the rotational force (moment along the longitudinal axis of the forearm) applied to hand‐held objects (Shim et al. 2004; Cole 2006; Olafsdottir et al. 2008; Cole et al. 2010; Parikh and Cole 2012). Furthermore, older adults were more variable in digit‐tip force production during a precision‐orientation task similar to inserting a key into a slot (Parikh and Cole 2012). Similar observations of force unsteadiness are reported in older adults working at low intensities of muscle contractions [mainly ≤20% of maximum voluntary contraction (MVC)] during isometric and slow anisometric tasks (Laidlaw et al. 2000, 2002; Enoka et al. 2003; Tracy et al. 2004, 2007). Increased finger force unsteadiness on isometric tasks with age has been linked to slowing on a dexterous manual task (Marmon et al. 2011a).

Long‐term practice and strength training paradigms that reduced the moment‐to‐moment variability in finger forces during isometric tasks also improved dexterous behavior in old age (Keen et al. 1994; Ranganathan et al. 2001; Kornatz et al. 2005; Marmon et al. 2011a,b). These improvements paralleled a reduction in motor unit discharge rate variability (Kornatz et al. 2005) and an increase in spinal motorneuron excitability assessed using H‐reflex (Ranganathan et al. 2001). Hence, improved finger force control and neural changes induced by repetition and/or increased excitability of the corticospinal tract may contribute to the improved dexterous behavior in older adults. This raises the possibility that improved fine motor performance following short‐term direct cortical stimulation in healthy and diseased individuals (Hummel et al. 2006, 2010; Tanaka et al. 2009; Zimerman et al. 2013) occurred through increased excitability of relevant neural circuitry (Nitsche and Paulus 2000). However, specific changes in the finger forces during grasp and manipulation resulting from a single session of anodal tDCS are not known.

To address these gaps, we investigated the effects of anodal stimulation (tDCS) of the scalp over the region of primary motor cortex (M1) representing the contralateral hand *in combination with motor practice* in healthy older adults on: (1) performance on functionally relevant Grooved Pegboard test; (2) the completion time during another object manipulation task, the ‘key‐slot’ task; (3) the fluctuations in forces applied to an object with a precision grip (thumb‐finger) during a ‘key‐slot’ task; and (4) the moment‐to‐moment force variability during an isometric precision grip task. Each participant practiced the Grooved Pegboard task while receiving anodal tDCS or sham (placebo) tDCS in two separate sessions (crossover design). Based on previous literature (Hummel et al. 2010; Zimerman et al. 2013), we hypothesized that anodal tDCS in combination with motor practice (anodal tDCS + MP) will lead to greater improvement and increased retention of the performance on the Grooved Pegboard test, and improve performance on our functionally relevant key‐slot task compared to sham tDCS with motor practice (sham tDCS + MP). Furthermore, we hypothesized that anodal tDCS + MP will improve force steadiness during performance of isometric and functionally relevant tasks compared to sham tDCS + MP, as anodal tDCS has been shown to enhance the effects of motor practice (Zimerman et al. 2013).

## Materials and Methods

### Subjects

Eight older adults (63–84 years; 75 ± 8 years [mean ±SD]; three females) served as participants. All participants self‐reported that the right hand was their preferred hand, and claimed to be free of the following: (1) injury or disease of the brain, (2) injury or disease affecting the hands or arms, (3) wrist or hand pain that requires daily prescription medication, (4) diabetes, (5) high blood pressure requiring medication, (6) sensory disturbances of the dominant arm, (7) corrected vision worse than 20/20, (8) history of heart disease, (9) implanted battery‐driven devices such as implanted pacemakers, defibrillators, infusion pumps, (10) family history of epilepsy, and (11) presence of metal in skull. All participants appeared to be aware of their surroundings and current events based on their responses to questions designed to screen for impaired cognitive status (Cole et al. 2010; Parikh and Cole 2012). Finally, to rule out undiagnosed sensory neuropathy, participant's ability to sense vibration applied to the distal interphalangeal joint of the right index finger was tested using the Rydel‐Seiffer graduated tuning fork (Arno Barthelmes, Tuttlingen, Germany). Those with a vibration threshold within the limits of published norms for their age (Martina et al. 1998) were invited to provide informed consent and participate in the study. Each subject completed a Transcranial Magnetic Stimulation (TMS) adult safety screen questionnaire to confirm their eligibility to participate in the TMS procedure (Keel et al. 2000). The University of Iowa Human Subject Internal Review Board approved the experiment and an informed consent was obtained from all subjects according to the Declaration of Helsinki.

### Apparatus

#### Key‐slot object

**Figure 1:**
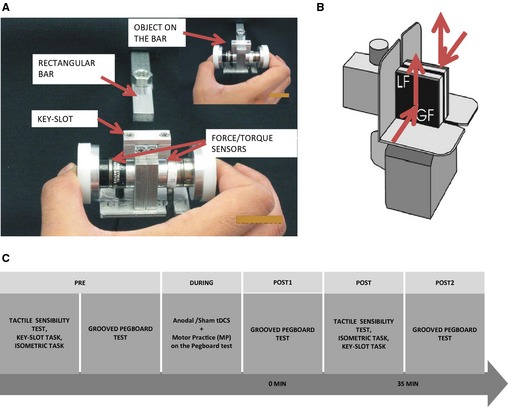
(A) Key‐slot object: Two force‐torque transducers mounted on the aluminum frame measured the thumb and index finger forces in Fx, Fy, and Fn axis during the key‐slot tasks. During the key‐slot task, participants attempted to fit the key‐slot (indicated by an arrow) on the object over the stationary rectangular bar (indicated by an arrow). Once the object fitted on the slot, participants slid it over the bar (inset figure). (B) Grip‐lift object: Strain gauges embedded in the gripping surfaces measured the vertical load force (LF) at contact surfaces of both digits. (C) Experimental procedure.

This novel object (100 g; Fig. 1A) has been previously described in Parikh and Cole (2012). It was instrumented with two‐six degree‐of‐freedom force/torque transducers (Nano Force/Torque System, ATI Industrial Automation, Apex, NC) on opposite sides of a small aluminum frame to measure the contact forces at the thumb and index finger. A flat plastic disc (33 mm diameter) covered with sandpaper (grit‐320) was attached to the outer surface of each transducer. The distance between the outer surfaces of the two pads was 7.4 cm. A rectangular keyway (‘slot’) was centered on the top of the object, which matched a rectangular aluminum bar on an adjacent table for the ‘key‐slot task’. The object rested on a table that had a narrow opening providing passage for the cables emerging from the transducers.

#### Grip‐lift object

The second test object (230 g; Fig. 1B) had two opposing contact surfaces (35 by 35 mm) parallel to each other, with a separation of 2.2 cm between the digit contact surfaces (Cole et al. 1999; Parikh and Cole 2013). The plates were covered with black sandpaper (grit‐320). Strain gauges integrated in the object measured the vertical tangential (load) force separately at both contact surfaces. An accelerometer (SenSyn SXL010G, Sunnyvale, CA) affixed to the object measured the vertical acceleration.

### Assessment

#### Tactile sensibility

Tactile thresholds were obtained from the distal volar pads of the index finger using Semmes–Weinstein pressure filaments (Smith Torque System, Menominee Falls, WI). We used a descending method of limits to establish a threshold. The index finger was tested approximately midway between the center of finger pad and the radial margin of the finger. A threshold was recorded for the smallest filament diameter that could be perceived on at least 70% of its applications.

#### Key‐slot task

The key‐slot object (Fig. 1A) was positioned on the table so that reaching for it required minimal shoulder/trunk motion. The height of the table or chair was adjusted so that the hand and the object were at the same level. The experimenter provided a brief demonstration of the task to the subjects. Subjects could see their hand as they performed this task but the experimenter did not comment on object orientation. Subjects were instructed to grasp the test object at a self‐selected speed using a precision grip with their thumb and index finger, lift the object to a height of approximately 30 cm above the table surface while maintaining neutral forearm rotation, and hold it steady at that elevation for 5 sec. This positioned the object roughly at the same height as the rectangular bar with its long axis perpendicular to the frontal plane of the subject. Their instructions then were to fit the object's slot onto the rectangular bar, without assistance from the opposite hand, and slide the object 3 cm until it met a rigid stop. There were no specific instructions regarding how to orient the object. Subjects performed the lift with a combination of elbow and shoulder flexion. They repeated the key‐slot task five times with approximately 10 sec between trials, before and after the stimulation.

#### Isometric precision grip task

Target forces equivalent to 5% and 10% of maximum precision force (MPF), and the subject's grip force signal were displayed as separate bright dots on the oscilloscope. Subjects were instructed to squeeze the grip‐lift object stabilized on the table using a double‐sided tape using a pulp‐to‐pulp precision of the index finger and thumb (Fig. 1B), and then maintain the target precision force for approximately 15 sec. Following this, the screen of oscilloscope was blocked using white cardboard. Subjects were instructed to continue to maintain the target precision force for another 15 sec. They performed three practice trials, followed by three trials each at 5% MPF and 10% MPF, before and after the stimulation. To measure the MPF, subjects pinched maximally with their thumb and index finger of the dominant hand against a standard clinical precision force gauge (B&L Engineering) supported by the examiner. They performed three trials, and the MPF was recorded during each of the three trials. The best of the three trials was used as the MPF.

#### Grooved Pegboard test

Subjects were tested for their fine manual performance using the Grooved Pegboard test (Marmon et al. 2011b). Subjects were instructed to insert pegs one by one into 25 different holes at their maximal speed using their dominant right hand. Each subject performed two Pegboard trials before the stimulation (pre), immediately after the stimulation (post1), and 35 min post stimulation (post2).

#### Experimental procedure

Subjects participated in two experimental sessions each lasting 2 h (Fig. 1C). After initial familiarization, subjects washed their hands with soap and water. They sat in front of the table associated with the specific task, with their dominant hand resting on their thigh. During each session, subjects were tested for tactile sensibility, and then were instructed to perform the key‐slot task, and the isometric precision grip task. Subjects were, then, tested on the Grooved Pegboard test. Following these baseline measurements, subjects received either anodal tDCS or sham tDCS to the primary motor cortex contralateral to the dominant hand, in combination with motor practice (tDCS + MP). Each subject received anodal tDCS + MP and sham tDCS + MP in two sessions separated by more than 5 days. During anodal and sham tDCS, subjects were instructed to practice the Pegboard test six times during the stimulation period (during; Fig. 1C). The two stimulation conditions were counterbalanced, that is, half of the subjects first received anodal tDCS + MP, while the other half received sham tDCS + MP during their first session. The experimenter, but not the subjects were aware of the order of this presentation. Immediately following the stimulation, subjects performed the Pegboard test. This was followed by tactile sensibility testing, performances of the key‐slot task, and isometric precision grip task. The order of the presentation of the key‐slot task, and the isometric precision grip task was counterbalanced across subjects. Subjects were again tested on the Pegboard test at the end of the session, which was 35 min after the completion of tDCS stimulation. Participants were explicitly asked at the end of second experimental session whether they could ascertain which session involved stimulation and which session involved sham stimulation.

#### Transcranial direct current stimulation

Transcranial direct current stimulation was applied via two conducting 25 cm^2^ saline‐soaked sponge electrodes. In a bipolar electrode montage, an anodal electrode on the scalp overlying the left primary motor cortex hand area and the cathodal electrode on the skin over the right supraorbital region were secured using elastic bands. An iontophoresis device (Chattanooga Group, Vista, CA) was used to apply constant current with square waveform at an intensity of 1 mA (current density: 0.04 mA/cm^2^; total charge: 0.048 C/cm^2^) for 20 min in the anodal tDCS group and for up to 30 sec in the Sham session. For anodal tDCS, the current was ramped up in 30 sec, administered for 20 min, followed by a 30‐sec ramp down period. Anodal tDCS using these parameters has been proven to increase the corticospinal excitability of the region under the stimulation without inducing side effects (Nitsche and Paulus 2001). For sham tDCS, the current was ramped up to 1 mA in 30 sec and then the stimulation device was turned off gradually by the experimenter. The effects of anodal tDCS on motor performance/skill acquisition are enhanced when it is applied in combination with motor training/learning (Reis et al. 2008, 2009; Zimerman et al. 2013). In our study, each participant practiced a motor task while receiving anodal tDCS and sham (placebo) tDCS in two separate sessions. The practice on the Pegboard test was started after the disappearance of physical skin sensation, in the form of mild tingling or burning sensations, which usually lasted for 30 sec following initiation of the stimulation.

Each experimental session began with an estimation of the site of tDCS anodal electrode placement using single‐pulse transcranial magnetic stimulation (TMS) technique. Subjects were comfortably seated in an adjustable chair with a head support, and the elbow flexed at ~90 degree allowing the hand to rest comfortably on the table. TMS was applied using a figure‐of‐eight coil with a 7 cm diameter (Magstim Company Ltd., Whitland, Dyfed, UK). The TMS coil was held tangential to the scalp and perpendicular to the presumed direction of the central sulcus, 45° from the midsagittal line, with the handle pointing backward (Mills et al. 1992). Using suprathreshold TMS pulses, we located the region of the left motor cortex that represented the right first dorsal interosseus muscle (FDI), which in turn corresponds to the hand ‘knob’ area in the primary motor cortex (Yousry et al. 1997). The position of the coil was adjusted until a reliable visible muscle twitch, in the form of isolated abduction of the contralateral index finger, was evoked (Fried et al. 2011). This location was marked on the scalp using a surgical marker pen, and acted as the site of anodal electrode placement following baseline measurements.

### Data analysis

Force and acceleration signals were acquired at 500 samples per second with a 16‐bit resolution, and analyzed with a personal computer running Datapac software (Datapac 2000 v 2.0 RUN Technologies, Mission Viejo, CA), except the performance on the Grooved Pegboard test.

#### Key‐slot task

We calculated the angles of the fingertip and thumb force (in degrees) measured independently in the proximal‐distal (horizontal) plane, and in the radial‐ulnar (vertical) plane in the local reference frame of the transducers (Parikh and Cole 2012).

$$\begin{array}{c} \text{Force angle in the vertical plane}\\ ={\text{tan}}^{-1}\left[\text{Fy}÷\text{Fn}\right]\times 360/2\pi \end{array}$$

$$\begin{array}{cc} \text{Force angle in the horizontal plane}\\ & ={\text{tan}}^{-1}\left[\text{Fx}÷\text{Fn}\right]\times 360/2\pi \end{array}$$

For the key‐slot manipulation task, we measured the mean and standard deviation of horizontal and vertical force angles at each digit for each subject from the contact of the object with the bar until the object was fitted on the bar. The event of contact between the object and bar was estimated from the object acceleration signal. Force signatures characteristic to the start/end of these phases made determining this phase relatively easy (Parikh and Cole 2012). We measured the time to articulate the object with the bar (Parikh and Cole 2012). Mean and standard deviation of grip force applied to the object were also computed. The mean grip force was defined as an average of the Fn forces applied to the object at each contact surface (i.e., {Fn_thumb_ + Fn_finger_} ÷ 2). For each of these measures, mean values across five trials were obtained for each subject before and after stimulation. The means were entered into repeated measures analysis of variance to determine the effects of stimulation (anodal, sham), time (pre, post) as within‐subject factors.

#### Isometric precision grip task

Consistent with previous studies that used a similar task, the force signal drifted about the target force during the performance of the task in absence of visual feedback (Tracy et al. 2007). This low‐frequency drift was removed using the dynamic demeaning function in the analysis software, which subtracted the mean of the signal calculated over a fixed time window that was passed over the data in increments of one data point. This function detrended force fluctuation around a mean of zero. Thus, the mean pinch force for the vision and no‐vision conditions was computed before the removal of drift (Tracy et al. 2007). Force steadiness of precision force was quantified by measuring standard deviation of the force (detrended) about the mean, that is, coefficient of variation (COV) of grip force during an 8‐sec segment during the constant force task under both vision and no‐vision conditions. To investigate the changes in grip force variability, repeated measures ANOVA was used with stimulation (anodal, sham), time (pre, post), vision (vision, no‐vision), and force (5% MVC, 10% MVC) as within‐subject factors.

#### Grooved Pegboard test

Performance on the Pegboard test was assessed by measuring the time (in seconds) required to perform the trial beginning when the subject started their hand movement and continuing until the last peg was inserted. We also counted the number of pegs dropped as ‘errors’. A dropped peg is defined as an unintentional drop of a peg from the time the subject attempts to pick up the peg from the tray until it is placed correctly in the hole. Separate repeated measures ANOVAs with stimulation (anodal, sham), and time (pre, during, post1, post2) as within‐subject factors were used to investigate the change in the Pegboard performance and the errors following stimulation.

All statistical analyses were performed using SPSS (IBM SPSS Statistics for Windows, Version 19.0; IBM Corp Armonk, NY). Huyn‐Feldt correction was applied in case of violation of sphericity assumption. Post hoc comparisons were performed using paired *t*‐test with appropriate Bonferroni corrections (adjusted *α*‐levels are stated when appropriate). All values in the text and figures represent group mean ± 1 standard error. Significance level for all statistical analyses was set at *α *= 0.05.

## Results

All participants completed the study with no adverse effects. Each old adult self‐reported mild tingling or burning skin sensations under the stimulating electrodes for the initial 30 sec or so of each anodal and sham stimulation period, followed by no sensation for the remainder of the stimulation period (McFadden et al. 2011). None of the participants were able to distinguish between anodal tDCS and sham tDCS.

### Grooved Pegboard test

**Figure 2:**
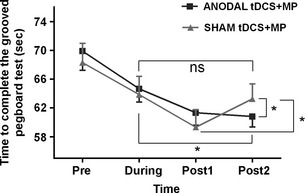
Performance on the Grooved Pegboard test before (pre), during, and following (post1, post2) anodal and sham stimulation. Asterisk indicates *P* < 0.0167. ns indicates nonsignificant.

Immediately following anodal and sham tDCS + MP (post1), subjects demonstrated improved performance on the Pegboard test as indicated by the reduction in time to insert 25 pegs one by one in the board at the maximum speed (Fig. 2). When performance was measured again 35 min later (post2), the observed improvement on the Pegboard test in the anodal tDCS + MP condition was retained, while that in the sham tDCS + MP condition deteriorated (Fig. 2; Significant Stimulation × Time interaction: *F*_1.99, 13.99 _= 5.43; *P* = 0.01; main effect of Time: *F*_3, 21 _= 63.22; *P* < 0.001). Post hoc analysis showed that following sham tDCS + MP, but not following anodal tDCS + MP, subjects were significantly slower in completing the Pegboard test 35 min later (paired *t*‐test post1 vs. post2: sham tDCS + MP: *t*_7_ = −5.242; *P* = 0.001; anodal tDCS + MP: *t*_7 _= 0.11; *P* > 0.3; adjusted *α*‐level = 0.0167). Subjects were significantly slower in completing the Pegboard test measured 35 min following sham tDCS + MP versus anodal tDCS + MP (paired *t*‐test post2: *t*_7_ = −3.5; *P* = 0.01; adjusted *α*‐level = 0.0167). There was no difference in the Pegboard test performance measured immediately following anodal tDCS + MP and sham tDCS + MP (paired *t*‐test post1: *P* > 0.2). Interestingly, following sham tDCS + MP versus anodal tDCS + MP, performance of subjects on the Pegboard test 35 min later was similar to that measured during the motor practice session (paired *t*‐test during vs. post2: sham tDCS + MP: *t*_7_ = 0.18; *P* = 0.86; anodal tDCS + MP: *t*_7 _= 5.65; *P* = 0.001; adjusted *α*‐level = 0.0167). There was no difference between the performances measured before subjects received anodal tDCS + MP and sham tDCS +MP (Baseline measures – paired *t*‐test: *t*_7_ = −0.240; *P* > 0.8). These findings suggest that anodal tDCS helped to retain the improvement in the performance gained from practicing the Pegboard task. There was no change in the number of dropped pegs (i.e., not more than two dropped pegs on average) following stimulation (Nonsignificant Stimulation × Time interaction; *P* > 0.5).

### Isometric precision grip task

For this task, subjects were instructed to produce constant force using pinch grip at 5% and 10% MVC with and without vision of their force and the target, as displayed on the oscilloscope. We pooled the data across various target force levels because the change in COV of grip force for 5% and 10% MVC force levels were not different following anodal and sham tDCS + MP (Nonsignificant Stimulation × Force interaction: *F*_1,7 _= 0.001; *P* > 0.9).

**Figure 3:**
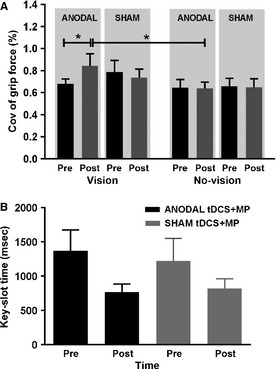
(A) Coefficient of variability (%) of grip force during the isometric precision grip force production task under vision and no‐vision conditions before (pre) and after (post) anodal and sham tDCS + MP. (B) Time to articulate the key‐slot over the stationary bar during the key‐slot task before (pre) and after (post) anodal and sham tDCS + MP. Asterisk indicates *P* < 0.025.

We found that a single session of anodal tDCS + MP increased the moment‐to‐moment variability in the grip force applied to the object (i.e., coefficient of variation [COV] of grip force), when compared with sham tDCS + MP (Fig. 3A; Significant Stimulation × Time interaction: *F*_1, 7 _= 5.6; *P* = 0.04; main effect of Vision: *F*_1, 7 _= 9.96; *P* = 0.016). Interestingly, post hoc paired *t*‐tests showed that, for the vision condition, the COV of grip significantly increased following anodal tDCS + MP, but not following sham tDCS + MP (Fig. 3A; anodal tDCS + MP: *t*_7 _= −2.85; *P* = 0.02; increased by 24%; sham tDCS + MP: *P* > 0.3; decreased by 6.6%; adjusted *α*‐level = 0.025). Furthermore, the COV of grip force for the vision condition was significantly greater than that for the no‐vision condition following anodal tDCS + MP but not sham tDCS + MP (Fig. 3A; anodal tDCS + MP: *t*_7 _= 2.8; *P* = 0.02; sham tDCS + MP: *P* > 0.1; adjusted *α*‐level = 0.025). This suggests that anodal tDCS + MP increased the COV of grip force more during vision condition than no‐vision condition.

### Key‐slot task

Subjects demonstrated similar reduction in the time to articulate the slot on key‐slot object with the stationary bar following anodal tDCS + MP and sham tDCS + MP (Fig 3B; main effect of Time: *F*_1, 7 _= 16.753; *P* = 0.03; Nonsignificant Stimulation × Time interaction: *F*_1, 7_ = 0.691; *P* > 0.4). That is, we observed 36% reduction in time to complete the key‐slot task following anodal tDCS + MP vs. 23% reduction in time following sham tDCS + MP).

Following anodal and sham tDCS + MP, subjects demonstrated no change in the moment‐to‐moment variability (standard deviation) in their force angles when trying to fit the slot on the object over the stationary bar (Nonsignificant Stimulation × Time interaction: *P* > 0.5; no main effect of Time: *P* > 0.05;). The standard deviations (SD) in force angles pooled across vertical and horizontal planes were 2.99° ± 0.15° and 2.56° ± 0.16°, before and after anodal tDCS + MP, respectively. The pooled SDs in force angles were 2.75° ± 0.27° and 2.54° ± 0.17°, before and after sham tDCS + MP, respectively. The change in the coefficient of variation of the magnitude of grip force measured when subjects attempted to articulate the object with the bar was similar following anodal tDCS + MP and sham tDCS + MP (Absence of Stimulation × Time interaction; *P* > 0.6). Similarly, there was no change in the magnitude of grip force applied to the object during the ‘key‐slot’ performance following anodal versus sham tDCS + MP (*P* > 0.3).

### Tactile sensibility

The old adults (OA) demonstrated normal‐for‐age tactile sensibility threshold, which did not change following anodal tDCS + MP and sham tDCS + MP. Before anodal and sham tDCS + MP, the tactile sensibility threshold was from 3.22 to 3.84 (mean: 3.39) and from 3.22 to 3.84 (mean: 3.39), respectively. Following anodal and sham tDCS + MP, the threshold was from 3.22 to 3.61 (mean: 3.32) and from 2.83 to 3.61 (mean: 3.37), respectively.

## Discussion

We investigated the effects of motor practice (MP) of the Pegboard task during tDCS over the primary motor cortex (M1) on the performance of functional grasp and manipulation tasks by healthy older adults. We also observed the digit‐tip forces applied to the object during a key‐slot task and an isometric precision grip task. The novel findings from our study are as follows: (1) Performance on the Grooved Pegboard task improved as a result of practice, regardless of the presence of anodal tDCS stimulation. However, anodal tDCS allowed elderly individuals to retain their improved performance on the Grooved Pegboard test for at least 35 min (longer delays were not tested), while practice‐only induced improvements (that is, sham tDCS + MP) decayed upon testing after the 35‐min retention delay, (2) In contrast to the apparent benefits of anodal stimulation for the functionally relevant task, anodal tDCS + MP appeared to negatively impact isometric precision grip force production under the vision condition, in the form of increased moment‐to‐moment variation in grip force magnitude.

Anodal tDCS over M1 in combination with motor practice facilitated retention of motor performance on a clinically relevant functional manual task (Grooved Pegboard) in healthy older adults, for at least a 35‐min retention duration. This suggests that a single session of short‐term (20 min) anodal stimulation of M1, when combined with physical practice, may be clinically used for enhancing retention of functional behavior in older age. The durability of this effect may extend well beyond the 35‐min retention delay interval that was tested here. Our current findings are consistent with Zimerman et al. (2013) that demonstrated the beneficial effect of anodal tDCS in combination with motor training in healthy older adults on skill acquisition and retention. They reported that anodal stimulation over M1 when healthy older adults were trained on a complex sequential finger‐tapping task improved performance during the stimulation period, and also allowed retention of this improvement up to 24 h post stimulation (Zimerman et al. 2013). Our result seems to extend the observation of improved retention of motor performance on a more natural and functionally relevant manual task.

The electrophysiological mechanisms underlying the retention of motor performance through anodal tDCS are not clear. One possibility is that anodal tDCS induced changes in glutamatergic receptor activity, which results in increased depolarization of membrane potential. The anodal stimulation‐mediated shift in the membrane potential modulates the activity of voltage‐dependent NMDA receptors, which is known to induce synaptic plasticity (Nitsche and Paulus 2000; Bütefisch et al. 2004). Therefore, it is plausible that the observed retention of motor performance in older adults is due to anodal tDCS increasing the NMDA‐receptor activation.

By contrast, anodal tDCS over M1 increased the moment‐to‐moment fluctuations in grip force during the isometric precision grip task in healthy older adults. One reason for increased variability following anodal tDCS could rest on how anodal tDCS influences underlying cortical activity. It is believed that the application of constant current alters spontaneous firing rates of neurons through changes in the ions bound in the neuronal cell membrane, and alterations in the transmembrane proteins (Zaghi et al. 2010). Specifically, anodal tDCS depolarizes the neuron making it more excitable. Churchland et al. (2006) have shown that firing patterns of neurons in primary motor cortex and premotor dorsal areas involved in movement preparation may contribute to the variability of peak hand velocity during repeated reaches to the same target (Churchland et al. 2006). In our study, anodal tDCS over M1 might have altered firing rates of the cortical neurons, which would have then caused an increase in fluctuations in the grip force during the isometric precision grip task performed after the 20‐min stimulation period.

An increase in force fluctuation during the isometric precision grip task following anodal tDCS + MP was observed when the older adults used visual feedback for more accurate production of isometric precision force (i.e., vision condition) but not when the visual display was obstructed (i.e., no‐vision condition). We observed no difference in force variability between the vision and no‐vision conditions before anodal tDCS, which is consistent with other reports (Christou 2005; Sosnoff and Newell 2006). Greater force variability under the visual feedback condition after receiving anodal tDCS could again result from increased cortical excitability following a single session of anodal tDCS. This increased excitability would have modulated the descending drive to spinal motor neurons involved in the performance of isometric precision grip task. The descending motor commands, along with other inputs from muscle spindles and tendon organs onto the alpha‐motor neurons, could modulate the variability of grip force (Enoka et al. 2003; Tracy et al. 2007). Furthermore, the burden of visuomotor processing (e.g., during our vision condition) increases oscillations in the descending drive, and/or alters the frequency content of common synaptic inputs to the active motor units leading to increased force fluctuations during the production of steady voluntary forces under conditions of visual force feedback (Tracy et al. 2007; Laine et al. 2013).

To summarize our findings, we observed that anodal tDCS over M1 when combined with motor practice allows healthy older adults to retain the gain in performance on a functionally meaningful manual task for an extended period. Moreover, a single 20‐min session of anodal tDCS can increase fluctuations in the grip force during an isometric precision grip force production task in healthy older adults. Further studies to assess the generalizability of the observed effects of anodal tDCS + MP on finger force control in larger older and younger population are needed.

## Conflict of Interest

The authors declare no conflict of interest.

## References

[phy2255-bib-0001] Bolognini, N. , and T. Ro . 2010 Transcranial magnetic stimulation: disrupting neural activity to alter and assess brain function. J. Neurosci. 30:9647–9650. 20660247 10.1523/JNEUROSCI.1990-10.2010 PMC6632835

[phy2255-bib-0002] Bütefisch, C. M. , V. Khurana , L. Kopylev , and L. G. Cohen . 2004 Enhancing encoding of a motor memory in the primary motor cortex by cortical stimulation. J. Neurophysiol. 91:2110–2116. 14711974 10.1152/jn.01038.2003

[phy2255-bib-0003] Christou, E. A. 2005 Visual feedback attenuates force fluctuations induced by a stressor. Med. Sci. Sports Exerc. 37:2126–2133. 16331140 10.1249/01.mss.0000178103.72988.cd

[phy2255-bib-0004] Churchland, M. M. , A. Afshar , and K. V. Shenoy . 2006 A central source of movement variability. Neuron 52:1085–1096. 17178410 10.1016/j.neuron.2006.10.034 PMC1941679

[phy2255-bib-0005] Cole, K. J. 2006 Age‐related directional bias of fingertip force. Exp. Brain Res. 175:285–291. 16738907 10.1007/s00221-006-0553-0

[phy2255-bib-0006] Cole, K. J. , D. L. Rotella , and J. G. Harper . 1999 Mechanisms for age‐related changes of fingertip forces during precision gripping and lifting in adults. J. Neurosci. 19:3238–3247. 10191336 10.1523/JNEUROSCI.19-08-03238.1999 PMC6782297

[phy2255-bib-0007] Cole, K. J. , K. M. Cook , S. M. Hynes , and W. G. Darling . 2010 Slowing of dexterous manipulation in old age: force and kinematic findings from the “nut‐and‐rod” task. Exp. Brain Res. 201:239–247. 19795110 10.1007/s00221-009-2030-z

[phy2255-bib-0008] Desrosiers, J. , R. Hébert , G. Bravo , and A. Rochette . 1999 Age‐related changes in upper extremity performance of elderly people: a longitudinal study. Exp. Gerontol. 34:393–405. 10433393 10.1016/s0531-5565(99)00018-2

[phy2255-bib-0009] Diermayr, G. , T. L. McIsaac , and A. M. Gordon . 2011 Finger force coordination underlying object manipulation in the elderly ‐ a mini‐review. Gerontology 57:217–227. 20224251 10.1159/000295921

[phy2255-bib-0010] Enoka, R. M. , E. A. Christou , S. K. Hunter , K. W. Kornatz , J. G. Semmler , A. M. Taylor , et al. 2003 Mechanisms that contribute to differences in motor performance between young and old adults. J. Electromyogr. Kinesiol. 13:1–12. 12488083 10.1016/s1050-6411(02)00084-6

[phy2255-bib-0011] Fried, P. J. , S. Elkin‐Frankston , R. J. Rushmore , C. C. Hilgetag , and A. Valero‐Cabre . 2011 Characterization of visual percepts evoked by noninvasive stimulation of the human posterior parietal cortex. Baker C. I., ed. PLoS One 6:e27204. 22087266 10.1371/journal.pone.0027204 PMC3210763

[phy2255-bib-0012] Galea, J. M. , and P. Celnik . 2009 Brain polarization enhances the formation and retention of motor memories. J. Neurophysiol. 102:294–301. 19386757 10.1152/jn.00184.2009 PMC2712265

[phy2255-bib-0013] Hummel, F. C. , B. Voller , P. Celnik , A. Floel , P. Giraux , C. Gerloff , et al. 2006 Effects of brain polarization on reaction times and pinch force in chronic stroke. BMC Neurosci. 7:73. 17083730 10.1186/1471-2202-7-73 PMC1636653

[phy2255-bib-0014] Hummel, F. C. , B. Voller , P. Celnik , A. Floel , P. Giraux , C. Gerloff , et al. 2010 Facilitating skilled right hand motor function in older subjects by anodal polarization over the left primary motor cortex. Neurobiol. Aging 31:2160–2168. 19201066 10.1016/j.neurobiolaging.2008.12.008 PMC2995492

[phy2255-bib-0015] Keel, J. C. , M. J. Smitha , and E. Wassermann . 2000 A safety screening questionnaire for transcranial magnetic stimulation. Clin. Neurophysiol. 112:720. 10.1016/s1388-2457(00)00518-6 11332408

[phy2255-bib-0016] Keen, A. , G. H. Yue , and R. M. Enoka . 1994 Training‐related enhancement in elderly humans in the control of motor output. J. Appl. Physiol. 77:2648–2658. 7896604 10.1152/jappl.1994.77.6.2648

[phy2255-bib-0017] Kornatz, K. W. , E. A. Christou , and R. M. Enoka . 2005 Practice reduces motor unit discharge variability in a hand muscle and improves manual dexterity in old adults. J. Appl. Physiol. (Bethesda, Md.: 1985), 98:2072–2080. 15691902 10.1152/japplphysiol.01149.2004

[phy2255-bib-0018] Laidlaw, D. H. , M. Bilodeau , and R. M. Enoka . 2000 Steadiness is reduced and motor unit discharge is more variable in old adults. Muscle Nerve 23:600–612. 10716772 10.1002/(sici)1097-4598(200004)23:4<600::aid-mus20>3.0.co;2-d

[phy2255-bib-0019] Laidlaw, D. H. , S. K. Hunter , and R. M. Enoka . 2002 Nonuniform activation of the agonist muscle does not covary with index finger acceleration in old adults. J. Appl. Physiol. 93:1400–1410. 12235041 10.1152/japplphysiol.00391.2002

[phy2255-bib-0020] Laine, C. M. , F. Negro , and D. Farina . 2013 Neural correlates of task‐related changes in physiological tremor. J. Neurophysiol. 110:170–176. 23596333 10.1152/jn.00041.2013

[phy2255-bib-0021] Marmon, A. R. , M. A. Pascoe , A. R. Marmon , M. A. Pascoe , R. S. Schwartz , and R. M. Enoka . 2011a Associations among strength, steadiness, and hand function across the adult life span. Med. Sci. Sports Exerc. 43:560–567. 20689447 10.1249/MSS.0b013e3181f3f3ab

[phy2255-bib-0022] Marmon, A. R. , J. R. Gould , and R. M. Enoka . 2011b Practicing a functional task improves steadiness with hand muscles in older adults. Med. Sci. Sports Exerc. 43:1531–1537. 21266932 10.1249/MSS.0b013e3182100439

[phy2255-bib-0023] Martina, I. S. J. , R. van Koningsveld , P. I. Schmitz , F. G. van der Meché , and P. A. van Doorn . 1998 Measuring vibration threshold with a graduated tuning fork in normal aging and in patients with polyneuropathy. J. Neurol. Neurosurg. Psychiatry 65:743–747. 9810949 10.1136/jnnp.65.5.743 PMC2170371

[phy2255-bib-0024] McFadden, J. L. , J. J. Borckardt , M. S. George , and W. Beam . 2011 Reducing procedural pain and discomfort associated with transcranial direct current stimulation. Brain stimul. 4:38–42. 21255753 10.1016/j.brs.2010.05.002 PMC3026574

[phy2255-bib-0025] Mills, K. R. , S. J. Boniface , and M. Schubert . 1992 Magnetic brain stimulation with a double coil: the importance of coil orientation. Electroencephalogr. Clin. Neurophysiol. 85:17–21. 1371739 10.1016/0168-5597(92)90096-t

[phy2255-bib-0026] Nitsche, M. A. , and W. Paulus . 2000 Excitability changes induced in the human motor cortex by weak transcranial direct current stimulation. J. Physiol. 527:633–639. 10990547 10.1111/j.1469-7793.2000.t01-1-00633.x PMC2270099

[phy2255-bib-0027] Nitsche, M. A. , and W. Paulus . 2001 Sustained excitability elevations induced by transcranial DC motor cortex stimulation in humans. Neurology 57:1899–1901. 11723286 10.1212/wnl.57.10.1899

[phy2255-bib-0028] Olafsdottir, H. B. , V. M. Zatsiorsky , and M. L. Latash . 2008 The effects of strength training on finger strength and hand dexterity in healthy elderly individuals. J. Appl. Physiol. (Bethesda, Md.: 1985), 105:1166–1178. 18687981 10.1152/japplphysiol.00054.2008 PMC2576040

[phy2255-bib-0029] Parikh, P.J. , and K.J. Cole . 2012 Handling objects in old age: forces and moments acting on the object. J. Appl. Physiol. (Bethesda, Md.: 1985), 112:1095–1104. 22241054 10.1152/japplphysiol.01385.2011

[phy2255-bib-0030] Parikh, P. J. , and K. J. Cole . 2013 Transfer of learning between hands to handle a novel object in old age. Exp. Brain Res. 227:9–18. 23595702 10.1007/s00221-013-3451-2

[phy2255-bib-0031] Ranganathan, V. K. , V. Siemionow , V. Sahgal , J. Z. Liu , and G. H. Yue . 2001 Skilled finger movement exercise improves hand function. J. Gerontol. A Biol. Sci. Med. Sci. 56:518–522. 10.1093/gerona/56.8.m518 11487606

[phy2255-bib-0032] Reis, J. , O. B. Swayne , Y. Vandermeeren , M. Camus , M. A. Dimyan , M. Harris‐Love , et al. 2008 Contribution of transcranial magnetic stimulation to the understanding of cortical mechanisms involved in motor control. J. Physiol. 586:325–351. 17974592 10.1113/jphysiol.2007.144824 PMC2375593

[phy2255-bib-0033] Reis, J. , H. M. Schambra , L. G. Cohen , E. R. Buch , B. Fritsch , E. Zarahn , et al. 2009 Noninvasive cortical stimulation enhances motor skill acquisition over multiple days through an effect on consolidation. Proc. Natl Acad. Sci. USA 106:1590–1595. 19164589 10.1073/pnas.0805413106 PMC2635787

[phy2255-bib-0034] Salthouse, T. A. 1988 The complexity of age x complexity functions: comment on charaess and campbell. J. Exp. Psychol. Gen. 117:425–428.

[phy2255-bib-0035] Shim, J. K. , B. S. Lay , V. M. Zatsiorsky , and M. L. Latash . 2004 Age‐related changes in finger coordination in static prehension tasks. J. Appl. Physiol. (Bethesda, Md.: 1985), 97:213–224. 15003998 10.1152/japplphysiol.00045.2004 PMC2832863

[phy2255-bib-0036] Smith, C. D. , G. H. Umberger , E. L. Manning , J. T. Slevin , D. R. Wekstein , F. A. Schmitt , et al. 1999 Critical decline in fine motor hand movements in human aging. Neurology 53:1458–1461. 10534251 10.1212/wnl.53.7.1458

[phy2255-bib-0037] Sosnoff, J. J. , and K. M. Newell . 2006 Aging, visual intermittency, and variability in isometric force output. J. Gerontol. B Psychol. Sci. Soc. Sci. 61:P117–P124. 16497955 10.1093/geronb/61.2.p117

[phy2255-bib-0038] Stagg, C. J. , G. Jayaram , D. Pastor , Z. T. Kincses , P. M. Matthews , and H. Johansen‐Berg . 2011 Polarity and timing‐dependent effects of transcranial direct current stimulation in explicit motor learning. Neuropsychologia 49:800–804. 21335013 10.1016/j.neuropsychologia.2011.02.009 PMC3083512

[phy2255-bib-0039] Tanaka, S. , T. Hanakawa , M. Honda , and K. Watanabe . 2009 Enhancement of pinch force in the lower leg by anodal transcranial direct current stimulation. Exp. Brain Res. 196:459–465. 19479243 10.1007/s00221-009-1863-9 PMC2700246

[phy2255-bib-0040] Tracy, B. L. , W. C. Byrnes , and R. M. Enoka . 2004 Strength training reduces force fluctuations during anisometric contractions of the quadriceps femoris muscles in old adults. J. Appl. Physiol. (Bethesda, Md.: 1985), 96:1530–1540. 14565966 10.1152/japplphysiol.00861.2003

[phy2255-bib-0041] Tracy, B. L. , D. V. Dinenno , B. Jorgensen , and S. J. Welsh . 2007 Aging, visuomotor correction, and force fluctuations in large muscles. Med. Sci. Sports Exerc. 39:469–479. 17473773 10.1249/mss.0b013e31802d3ad3

[phy2255-bib-0042] Yousry, T. A. , U. D. Schmid , H. Alkadhi , D. Schmidt , A. Peraud , A. Buettner , et al. 1997 Localization of the motor hand area to a knob on the precentral gyrus ‐ A new landmark. Brain 120:141–157. 9055804 10.1093/brain/120.1.141

[phy2255-bib-0043] Zaghi, S. , M. Acar , B. Hultgren , P. S. Boggio , and F. Fregni . 2010 Noninvasive brain stimulation with low‐intensity electrical currents: putative mechanisms of action for direct and alternating current stimulation. Neuroscientist 16:285–307. 20040569 10.1177/1073858409336227

[phy2255-bib-0044] Zimerman, M. , M. Nitsch , P. Giraux , C. Gerloff , L. G. Cohen , and F. C. Hummel . 2013 Neuroenhancement of the aging brain: restoring skill acquisition in old subjects. Ann. Neurol. 73:10–15. 23225625 10.1002/ana.23761 PMC4880032

